# Photopic and scotopic spatiotemporal tuning of adult zebrafish vision

**DOI:** 10.3389/fnsys.2015.00020

**Published:** 2015-03-04

**Authors:** Nadine Hollbach, Christoph Tappeiner, Anna Jazwinska, Volker Enzmann, Markus Tschopp

**Affiliations:** ^1^Department of Ophthalmology, Inselspital, University of BernBern, Switzerland; ^2^Department of Ophthalmology, Basel University HospitalBasel, Switzerland; ^3^Department of Biology, University of FribourgFribourg, Switzerland

**Keywords:** adult zebrafish, contrast sensitivity, optokinetic response, spatiotemporal vision, photopic, scotopic

## Abstract

Sensitivity to spatial and temporal patterns is a fundamental aspect of vision. Herein, we investigated this sensitivity in adult zebrafish for a wide range of spatial (0.014 to 0.511 cycles/degree [c/d]) and temporal frequencies (0.025 to 6 cycles/s) to better understand their visual system. Measurements were performed at photopic (1.8 log cd m^−2^) and scotopic (−4.5 log cd m^−2^) light levels to assess the optokinetic response (OKR). The resulting spatiotemporal contrast sensitivity (CS) functions revealed that the OKR of zebrafish is tuned to spatial frequency and speed but not to temporal frequencies. Thereby, optimal test parameters for CS measurements were identified. At photopic light levels, a spatial frequency of 0.116 ± 0.01 c/d (mean ± SD) and a grating speed of 8.42 ± 2.15 degrees/second (d/s) was ideal; at scotopic light levels, these values were 0.110 ± 0.02 c/d and 5.45 ± 1.31 d/s, respectively. This study allows to better characterize zebrafish mutants with altered vision and to distinguish between defects of rod and cone photoreceptors as measurements were performed under different light conditions.

## Introduction

Contrast sensitivity (CS) reflects the lowest contrast at which an image can be discriminated from its background (West et al., 2002). CS is highly susceptible to defects in vision; therefore, a decrease in CS may be the first measurable functional alteration of an ocular disease (Owsley, 2003).

Measuring CS at different spatial and temporal frequencies provides spatiotemporal contrast sensitivity functions (CSFs; Schade, 1956; Enroth-Cugell and Robson, 1966). Generally, their curve resembles an inverted U-shape; CS is maximized at intermediate spatial and temporal frequencies and is reduced at higher and lower frequencies. This implies that vision is tuned in space and time (Umino et al., 2008). As temporal frequency is the result of spatial frequency multiplied by its speed, vision cannot be tuned to each of these three parameters simultaneously and remain independent of the other parameters (Umino et al., 2008). CS changes with light levels (Kelly, 1972; Hess and Nordby, 1986; Umino et al., 2008). The change from cone to rod vision may additionally involve a change in the temporal tuning preference from stimulus speed (at photopic light levels) to the temporal frequency (at scotopic light levels), which has been observed in mice (Umino et al., 2008).

The diurnal zebrafish (Danio rerio) is a popular model in visual system research, in part due to its relatively large eyes and cone-rich retina (Fadool and Dowling, 2008). There are a significant number of disease models involving larval zebrafish (Del Bene and Wyart, 2012). Furthermore, adult zebrafish have become increasingly important as a model for retinal degeneration and regeneration (Brockerhoff and Fadool, 2011; Tappeiner et al., 2013). Nevertheless, in zebrafish, CS has been explored only under selected conditions at photopic light levels (Maaswinkel and Li, 2003; Rinner et al., 2005; Tappeiner et al., 2012). Therefore, the aim of this work was to study CSFs and tuning properties of adult zebrafish in detail at both photopic and scotopic light levels. To determine CS, the optokinetic response (OKR) was assessed in an established two-alternative, forced-choice psychophysical procedure (Tappeiner et al., 2012). Because this method is widely used, our results can be readily compared to the results of other species (Gaillard et al., 2008; Umino et al., 2008; Franco et al., 2009).

## Materials and methods

### Animals

Wild type zebrafish (Danio rerio) of the AB (Oregon) strain aged 9–12 months were used. The fish were raised under standard conditions (Westerfield, 1995; Brand et al., 2002) in water at approximately 26.5° Celsius under a 14/10 h light/dark cycle. The measurements were performed at room temperature (approximately 20° Celsius). Before testing visual function at low light levels, the fish were dark-adapted for a minimum of 30 min. To facilitate the placement of the fish in the examination chamber, the fish were briefly sedated in 0.1% Tricaine (Sigma-Aldrich, Buchs, Switzerland). The fish were then irrigated with fresh water and were awake during the subsequent experiments (Tappeiner et al., 2012). All experimental research on the animals was approved by the governmental authorities (Cantonal Veterinary Office, Switzerland) and adhered to the Association for Research in Vision and Ophthalmology (ARVO) Statement for the Use of Animals in Ophthalmic and Vision Research.

### Measuring contrast sensitivity in adult zebrafish

CSFs were measured in adult zebrafish using a commercially available optomotor device originally designed for mice (OptoMotry®, Cerebral Mechanics, Lethbridge, AB, Canada) but modified for measuring zebrafish, as previously described by our group (Tappeiner et al., 2012). In contrast to mice, in which head and body movements are assessed (commonly referred to as the optomotor response), the relatively large eyes of the zebrafish allow for the direct observation of eye movements and therefore to assess the so-called OKR. The fish were positioned in an examination chamber, which was designed to allow a constant flow of fresh water (Tappeiner et al., 2012). Virtual three-dimensional sine-wave gratings of variable spatial frequencies and/or speed were presented to adult zebrafish on four monitors that formed the walls of a cuboidal chamber (length × width × height = 39 × 39 × 32.5 cm). The eye movements were recorded with a Sony DCR-HC26 Handycam and analyzed on a live video monitor by the same observer for all experiments. Using the staircase strategy of the OptoMotry® device (OptoMotry® version 1.7.0), the contrast of the grating was decreased until the animal no longer responded. The contrast was then changed several times to identify the threshold. A correct response was defined as three or more consecutive saccades in the correct direction. Gratings below the CS of the zebrafish resulted in random eye movements similar to the random eye movement pattern observed with stationary gratings (Tappeiner et al., 2012). To allow a certain comparison to CSFs in other species, we applied the same parameters that Umino et al. used for mice (Umino et al., 2008). First, to investigate the dependency of the CS on the luminance level, CSFs were measured at an 8-log unit rang of luminance between 1.8 log cd m^−2^ (70 cd m^−2^) and −6.3 log cd m^−2^. The light levels were attenuated with cylindrical neutral density filters (R211 0.9ND, LEE Filters, Hampshire, UK) that encircled the examination chamber of the zebrafish from the bottom to the top of the optomotor device. These measurements were performed at a constant temporal frequency of 1.5 cycles/second (c/s) for different spatial frequencies ranging from 0.014 to 0.511 cycles/degree (c/d). To further characterize CSFs at scotopic and photopic light levels, CS was evaluated at spatial frequencies ranging from 0.014 to 0.511 cycles/degree (c/d)—namely 0.014, 0.031, 0.064, 0.128, 0.236, 0.383, and 0.511 c/d—and at speeds of rotation ranging from 0.4 to 24 degrees/second (d/s)—namely, 0.4, 0.75, 1.5, 3, 6, 12, and 24 d/s. These parameters corresponded to temporal frequencies ranging from 0.025 to 6 c/s—namely, 0.025, 0.05, 0.1, 0.2, 0.4, 0.75, 1.5, 3, and 6 c/s. These measurements were performed at luminance levels of 1.8 log cd m^−2^ and −4.5 log cd m^−2^. The same six zebrafish were used throughout the study and for each set of parameters.

### Statistics and analysis

As we observed the typical inverted U-shape of the CSFs in our study, we evaluated CS with the linear systems theory of Watson (De Valois and De Valois, 1980; Watson, 1986). This proved also useful to analyze CSFs of mice with the OptoMotry® setup (Umino et al., 2008). Umino et al. have derived the following equation to represent the overall CSF (*G*) (Umino et al., 2008). It models visual information processing with a series of spatio- temporal filters having bandpass and/or low-pass characteristics:

(1) $$G(f_{s},f_{t},s_{p})=\frac{kf_{t}}{{\left(1+{\left(\frac{f_{s}}{f_{so}}\right)}^{2}\right)}^{2}\left(1+{\left(\frac{f_{t}}{f_{to}}\right)}^{2}\right)\left(1+{\left(\frac{s_{p}}{s_{po}}\right)}^{2}\right)}$$

where *k* is a scaling factor, and *f_t_* is the temporal frequency of the stimulus. *Gf_s_*, *Gf_t_*, and *Gs_p_* are low-pass filter functions relating CS to spatial frequency (*f_s_*, cycles per degree), temporal frequency (*f_t_*, cycles per second), and grating speed (*s_p_*, degrees per second). To further improve the fitting of our results and to account for the fact that CSFs do not necessarily level at a CS of 1 in all setups, an additional parameter (*b*) was added:

(2) $$G(f_{s},f_{t},s_{p})=b+\frac{kf_{t}}{{\left(1+{\left(\frac{f_{s}}{f_{so}}\right)}^{2}\right)}^{2}\left(1+{\left(\frac{f_{t}}{f_{to}}\right)}^{2}\right)\left(1+{\left(\frac{s_{p}}{s_{po}}\right)}^{2}\right)}$$

Curve fitting for Figures 2, 3 was accomplished with the above equation (2) using GraphPad Prism software (version 5.0f; GraphPad Software, Inc., La Jolla, CA, USA), including automated fitting of unknown parameters in the equation (*b*, *k*, *f_so_*, *f_to_*, *s_po_*) for each individual curve automatically (nonlinear regression, robust fit, automated determination of parameters for a best-fit curve).

**Figure 1:**
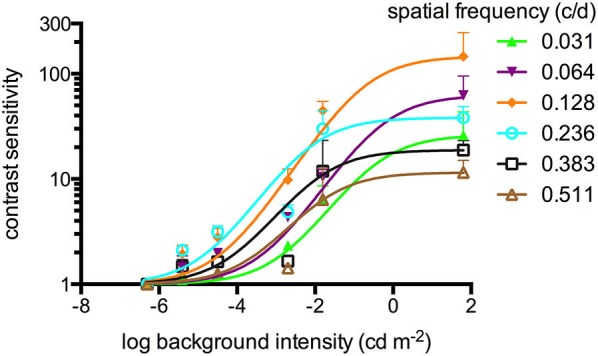
**Contrast sensitivities (sigmoidal curve fitting) of adult zebrafish (*n* = 6 animals) at different light intensities at a constant temporal frequency of 1.5 cycles/second (c/s)**. An optokinetic response (OKR) was not observed at the lowest light intensity tested (−6.3 log cd m^−2^). Above his light level, increasing contrast sensitivity (CS) was observed for increasing luminance at all tested spatial frequencies. Error bars indicate the standard deviation.

**Figure 2:**
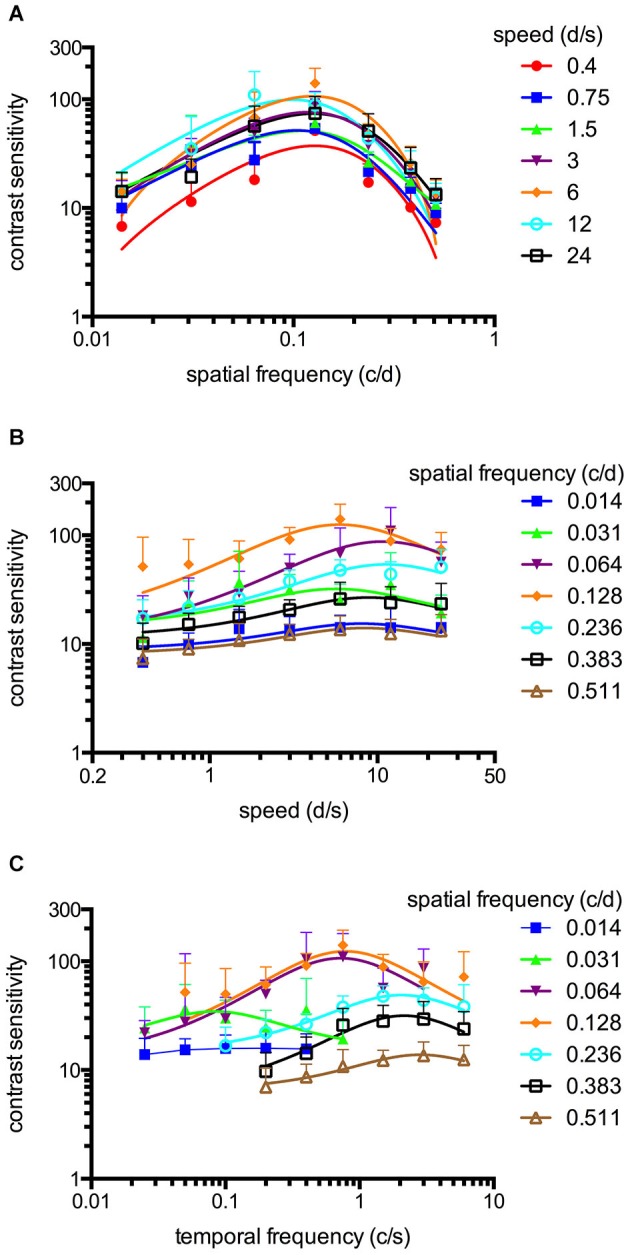
**Spatiotemporal contrast sensitivity functions (CSFs) of adult zebrafish at a constant photopic light level (1.8 log cd m^−2^, *n* = 6 animals). (A)** CSFs of varying speed measured at spatial frequencies ranging from 0.014 to 0.511 cycles/degree (c/d). **(B)** CSFs measured at speed levels ranging from 0.4 to 24 degrees/second (d/s). **(C)** CSFs measured at temporal frequencies ranging from 0.025 to 6 cycles/second (c/s). Error bars indicate the standard deviation.

**Figure 3:**
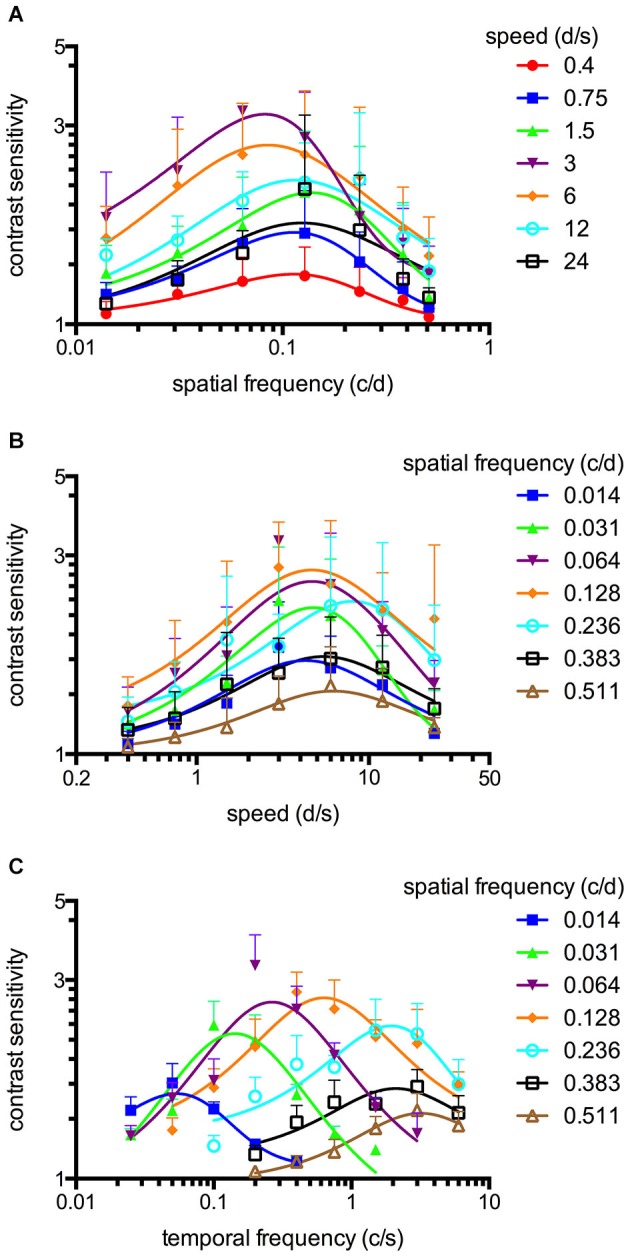
**Spatiotemporal contrast sensitivity functions (CSFs) of adult zebrafish at a constant scotopic light level (−4.5 log cd m^−2^, *n* = 6 animals). (A)** CSFs of varying speed measured at spatial frequencies ranging from 0.014 to 0.511 c/d. **(B)** CSFs measured at speed levels ranging from 0.4 to 24 d/s. **(C)** CSFs measured at temporal frequencies ranging from 0.025 to 6 cycles/second (c/s). Error bars indicate the standard deviation.

**Figure 4:**
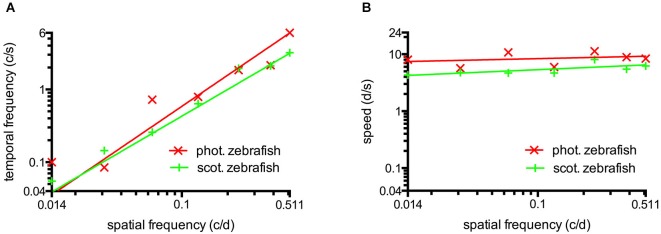
**(A)** Temporal frequency eliciting maximal CS as a function of spatial frequency. To elicit maximum CS, an increasing temporal frequency is necessary at higher spatial frequencies. This result indicates the absence temporal frequency tuning. **(B)** The speed of the rotating grating eliciting maximal CS as a function of spatial frequency. Maximum CS was triggered by a similar speed for a broad range of different spatial frequencies indicating tuning for speed at photopic and scotopic luminance levels. Straight lines were calculated by logarithmic nonlinear fitting.

**Figure 5:**
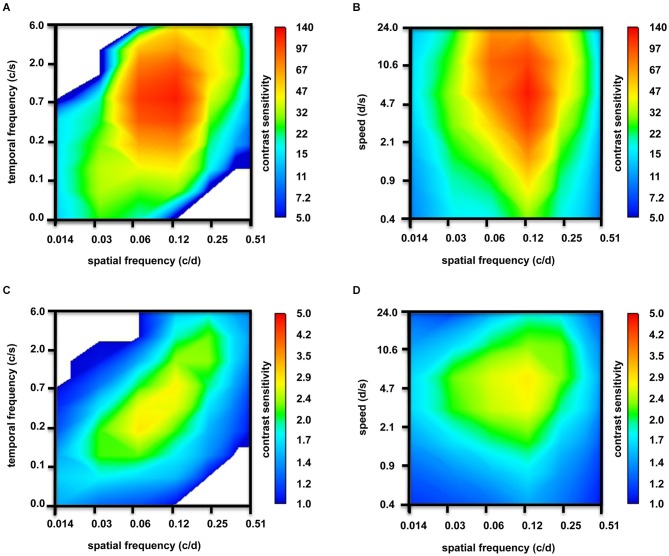
**Contour plots depicting tuning properties of adult zebrafish (*n* = 6 animals). (A)** At photopic light levels, strong tuning to spatial frequencies, but no temporal frequencies tuning was observed. **(B)** In addition to tuning to spatial frequencies, some tuning to speed is observed at photopic light levels. **(C)** At scotopic light levels, the tuning to spatial frequencies was weaker. Again, there was no temporal frequencies tuning. **(D)** However, clear tuning to speed was observed at scotopic light levels.

The maxima of the fitted curves correspond to the best CS. The corresponding value of the abscissa directly represents ideal settings to measure visual functions (data of Figures 2A,B, 3A,B) and were used to draw Figure 4 (data of Figures 2B,C, 3B,C). Means ± standard deviation (SD) were calculated using the ideal settings of the individual curves. The fitted curves also served to draw the contour plots (Figure 5). Six zebrafish were used for each set of parameters.

## Results

### Contrast sensitivity at different luminance levels

An OKR was not observed at the lowest light intensity (−6.3 log cd m^−2^, Figure 1). Starting at −5.4 log cd m^−2^, increasing CS was observed for increasing luminance at all tested spatial frequencies (Figure 1). At photopic light levels, a maximal CS of 140.8 ± 52.5 (mean ± SD) was observed at a spatial frequency of 0.128 c/d and a grating speed of 6 d/s, which corresponded to a temporal frequency of 0.75 c/s (Figure 2). In contrast, at scotopic light levels, a maximal CS of 3.44 ± 0.58 was observed at a spatial frequency of 0.064 c/d and a grating speed of 3 d/s, which corresponded to a temporal frequency of 0.2 c/s (Figure 3).

Equation 2 proved very useful to analyze the CSFs (Figures 2A,B, 3A,B) and the fitted curves allowed to calculate a general best spatial frequency and speed: At photopic light levels, a spatial frequency of 0.116 ± 0.01 c/d (mean ± SD) and a grating speed of 8.42 ± 2.15 d/s was determined; at scotopic light levels, these values were 0.110 ± 0.02 c/d and 5.45 ± 1.31 d/s, respectively. Interestingly, the general optimal spatial frequency is similar at photopic and scotopic light levels. However, at scotopic light levels, the highest CS is obtained with lower spatial frequencies (0.082 and 0.084 c/d at a speed of 3 or 6 d/s respectively, Figure 3A). This is also reflected by the fact that the single best CS is achieved with a spatial frequency of 0.064 c/d. As the maxima of the temporal frequency change with increasing spatial frequency (Figures 2C, 3C), it is not possible to calculate a general best temporal frequency (i.e., zebrafish vision is not tuned to temporal frequency).

### Spatiotemporal contrast sensitivity functions of adult zebrafish

CSFs were measured over a wide range of speed, spatial and temporal frequencies at photopic and scotopic light levels. The results are summarized in Figure 2 (photopic conditions, 1.8 log cd m^−2^, *n* = 6 animals for all conditions) and Figure 3 (scotopic conditions, −4.5 log cd m^−2^, *n* = 6 animals for all conditions).

### Tuning of the adult zebrafish photopic and scotopic vision

The maxima of the CSFs are at a very similar spatial frequency (Figures 2A, 3A). This clearly indicates tuning for spatial frequency with a maximum at 0.116 ± 0.01 c/d (mean ± SD) at photopic light levels and 0.110 ± 0.02 c/d at scotopic light levels. Furthermore, CSFs are tuned to speed at both bright and dim light levels (Figures 2B, 3B), as indicated by the constant peaks in the CS at a grating speed of 8.42 ± 2.15 d/s (mean ± SD) at photopic and 5.45 ± 1.31 d/s at scotopic light levels; regardless of the tested spatial frequency. In contrast, CSFs migrate along the abscissa when plotted against the temporal frequency at bright and dim light levels (Figures 2C, 3C). A similar pattern has been observed by Rinner et al. for larval zebrafish (tested at photopic conditions) (Rinner et al., 2005). This result implies the absence of temporal frequency tuning. To better illustrate the tuning to speed or temporal frequency, the speed/temporal frequency that elicited maximal CS was plotted for the tested spatial frequencies (this basically represents the maxima of the fitted curves of Figures 2A,B, 3A,B). As zebrafish vision is not tuned to temporal frequency, the temporal frequency that elicits maximal CS increases with increasing spatial frequency (Figure 4A). In contrast, the speed that induces maximum CS is fairly constant both at photopic and scotopic light levels, as would be expected for speed tuning (Figure 4B).

In Figure 5, the contour plots underlay the above-mentioned CSFs: at bright light levels, strong tuning to spatial frequencies (Figures 5A,B) and, to some degree, to speed (Figure 5B) is observed; however, temporal frequencies tuning is not observed (Figure 5A). At scotopic light levels, the tuning to spatial frequencies is weaker (Figures 5C,D). Again, we did not observe temporal frequencies tuning (Figure 5C), but tuning to speed was clear (Figure 5D).

## Discussion

The sensitivity to spatial and temporal patterns is a fundamental aspect of the visual system (Wandell, 1995). Here, we explore this sensitivity by presenting a wide range of spatiotemporal CSFs to adult zebrafish, a model organism with growing importance in visual science. Measurements were performed at photopic and scotopic light levels. To our knowledge, this is the first description of such measurements at low light levels in zebrafish. We employed the same OptoMotry® setup that was used by Umino et al. to establish these measurements in mice (Umino et al., 2008). This arrangement allows for the comparison of CSFs and visual tuning between mice and zebrafish.

In mammals and humans, CS increases with brighter light levels (da Silva Souza et al., 2011). This increase of the CS (and plateau at brighter light levels) was also observed in our study (Figure 1). With our setup measurements above a luminance of 1.8 log cd m^−2^ are not possible. However, studies in larval zebrafish suggest a stable CS above a luminance of 0.47 log cd m^−2^ (Rinner et al., 2005).

Interestingly, CS at scotopic light levels is similar in mice and zebrafish, with mice reaching a slightly higher CS of 5.3 at −4.5 log cd m^−2^ (Umino et al., 2008) than zebrafish. Humans have a considerably higher maximal CS of 80 at dim light levels (Hess and Nordby, 1986). At photopic levels, zebrafish and humans have a much higher CS than mice (Hess and Nordby, 1986; Umino et al., 2008). One reason for this difference may be that mice, as nocturnal animals, have a rod dominant retina with a rod-cone ratio of 97:3 in the central and peripheral retina (Carterdawson and Lavail, 1979). On the other hand, zebrafish, as a diurnal species, are functionally cone dominant (rod-cone ratio of approximately 65:35; data not shown) (Branchek, 1984; Bilotta and Saszik, 2001). Human retinas contain more rods than cones (rod-cone ratio 95:5), which is similar to mice; however, the fovea, as the central area, is primarily populated by cones (Wikler and Rakic, 1990). Compared with rods, cones are less sensitive to light but work best and most rapidly under photopic conditions due to their rapid receptor recovery following exposure to bright light (Wang and Kefalov, 2011). Accordingly, Lundh et al. (1983) showed that the density of cone photoreceptors is responsible for the high CS of humans under photopic conditions. Therefore, humans share the abilities of night vision with mice and other nocturnal animals but their vision in bright light is much better than the one of mice and even of zebrafish.

CS is highest for intermediate spatial and temporal frequencies/speeds (da Silva Souza et al., 2011), i.e., vision is tuned in space and time. This tuning results in a characteristic inverted U-shape form of the CSFs. Although this bandpass profile is observed in all species at photopic light levels (da Silva Souza et al., 2011), conflicting results exist regarding spatial CS at scotopic light levels. Some authors describe a low-pass function (Derefeldt et al., 1979; Bilotta and Powers, 1991; Benedek et al., 2003; Umino et al., 2008; Northmore, 2010) whereas other studies claim that a bandpass profile is retained at scotopic light levels (Hess and Nordby, 1986). The change from bandpass to low-pass behavior may be caused by spatial and temporal summation of photons, which acts to increase sensitivity in dim light (van Hateren, 1992, 1993; Warrant, 1999). Warrant developed a model to show that this low-pass shape as spatial and temporal summations is extremely useful for night vision but that their contributions depend a great deal on the design of the eye and the lifestyle of the nocturnal animal (Warrant, 1999). Nocturnal insects, for example, have superposed eyes, improving photon capture but leading to a loss of spatial resolution. The change from bandpass to low-pass shape does not seem to take place in adult zebrafish, as their CSFs showed bandpass characteristics at both high and low light levels (Figures 2, 3).

We observed the highest CS at intermediate spatial frequencies of 0.116 c/d at photopic light levels and 0.11 c/d at scotopic light levels. This preferred spatial frequency is similar to that observed in mice (Umino et al., 2008). In contrast, the preferred spatial frequency of the human visual system is considerably higher (approximately 2–6 c/d under photopic conditions) (Hess and Nordby, 1986; da Silva Souza et al., 2011). The reason for these differences in the preferred spatial frequency is still unclear. Theoretically, the mechanisms underlying spatial tuning may originate in the retina and/or in higher levels of the visual system (Umino et al., 2008). Da Silva Souza et al. noted that some features of CSF, such as the band-pass shape, are shared by all species, whereas other features vary among species (da Silva Souza et al., 2011). These variations depend on different morphological organizations (eye optics, photoreceptor distribution in retina, density of retinal neurons, post-receptoral procession) and result in different CS peaks, optimal spatial frequencies and visual acuities (Hughes, 1977; Jacobs et al., 1982). One intriguing explanation for the difference in preferred spatial frequency of humans and small animals is the difference in the size of the eye (Fite and Rosenfieldwessels, 1975; Hughes, 1977). For geometric reasons, a larger eye leads to a larger retinal image, resulting in better visual acuity if photoreceptor spacing is comparable (Haug et al., 2010; da Silva Souza et al., 2011; Tappeiner et al., 2012). Accordingly, if the size of the receptive fields in the retina remains constant, a larger eye also directs a higher preferred spatial frequency. This correlation would imply that the preferred spatial frequency is defined by the size of the eye and the receptive fields—which may, to a certain degree, be adapted to the needs of the animal—and not by higher-level visual system functions. Likewise, the tendency towards tuning to lower spatial frequencies at lower light levels, as observed in some studies (Ransomhogg and Spillmann, 1980; Umino et al., 2008), could be explained by the increasing central diameter of receptive fields at low light levels (Ransomhogg and Spillmann, 1980). This increase has been attributed to a decrease in lateral inhibition (Spillmann, 2006). In our study we did not observe a clear shift towards lower spatial frequencies at scotopic light levels- however, the best CS were obtained with lower spatial frequencies at scotopic light levels (Figure 3A).

In addition to spatial tuning, vision is also temporally tuned. Generally, vision can not be tuned to temporal frequency and speed at the same time under a specific test condition (e.g., one luminance level) given that temporal frequency is the result of spatial frequency multiplied by its speed. In mice, the tuning preference changes from speed to temporal frequency after the transition from cone to rod vision (Umino et al., 2008). We did not observe this shift in adult zebrafish; rather, vision in zebrafish revealed to be tuned to speed at bright as well as dim light levels. It is unclear why the tuning preference changes in one species but not the other. The measurements were performed at the same light levels, and the transition from cone to rod vision occurs at a similar light level in zebrafish, mice, and humans (Ren et al., 2002). One difference in the study design was that the optomotor response (OMR) was analyzed in mice, whereas in zebrafish, the OKR was analyzed, which may depend more on speed than the OMR. Alternatively, speed is perhaps more important to zebrafish than to mice. E.g., recognizing speed is important for prey capture (Ewert et al., 2001); and zebrafish live in a faster-moving habitat (water), which makes stabilization to the surroundings more difficult. Another possibility is that the nocturnal mice have a more specialized night vision. To the best of our knowledge, no data about this change in tuning preference in humans is available. However, there is some evidence that no such shift occurs in primates given that motion-sensitive neurons in the primary visual cortex do not change their firing properties with changing light levels (Duffy and Hubel, 2007).

The obtained CSFs in this study allow for the identification of ideal test parameters for visual acuity and CS tests. Specifically, a spatial frequency of 0.116 c/d and a grating speed of 8.42 d/s are optimal for photopic light levels, whereas a spatial frequency of 0.110 c/d and a grating speed of 5.45 d/s are optimal for scotopic light levels. Measurements at low light levels enable the differentiation of defects in rod and cone photoreceptors. In conclusion, this study increases our understanding of basic aspects of the zebrafish visual system, in part by describing the tuning of zebrafish vision. Furthermore, these results lay the basis for optimal testing of adult zebrafish visual acuity and CS and may allow for improved characterization and identification of mutants with altered vision.

## Author contributions

Conceived and designed the experiments: MT NH CT VE AJ. Performed the experiments NH. Analyzed the data CT MT NH. Wrote the paper MT NH CT. All authors contributed to and approved the final manuscript.

## Conflict of interest statement

The authors declare that the research was conducted in the absence of any commercial or financial relationships that could be construed as a potential conflict of interest.

## References

[B1] Benedek G. Benedek K. Keri S. Letoha T. Janaky M. (2003). Human scotopic spatiotemporal sensitivity: a comparison of psychophysical and electrophysiological data. Doc. Ophthalmol. 106, 201–207. 10.1023/A:1022548013313 12678286

[B2] Bilotta J. Powers M. K. (1991). Spatial contrast sensitivity of goldfish–mean luminance, temporal frequency and a new psychophysical technique. Vision Res. 31, 577–585. 10.1016/0042-6989(91)90108-h 1843762

[B3] Bilotta J. Saszik S. (2001). The zebrafish as a model visual system. Int. J. Dev. Neurosci. 19, 621–629. 10.1016/s0736-5748(01)00050-8 11705666

[B4] Branchek T. (1984). The development of photoreceptors in the zebrafish, brachydanio-rerio .II. Function. J. Comp. Neurol. 224, 116–122. 10.1002/cne.902240110 6715575

[B5] Brand M. Granato M. Nuesslein-Volhard C. (2002). “Keeping and raising zebrafish,” in Zebrafish–A Practial Approach, eds Nuesslein-Volhard C. Dahm R. (Oxford, UK: Oxford University Press), 7–37.

[B6] Brockerhoff S. E. Fadool J. M. (2011). Genetics of photoreceptor degeneration and regeneration in zebrafish. Cell. Mol. Life Sci. 68, 651–659. 10.1007/s00018-010-0563-8 20972813 PMC3029675

[B7] Carterdawson L. D. Lavail M. M. (1979). Rods and cones in the mouse retina .I. Structural-analysis using light and electron-microscopy. J. Comp. Neurol. 188, 245–262. 10.1002/cne.901880204 500858

[B30] da Silva Souza G. Gomes B. D. Silveira L. C. L. (2011). Comparative neurophysiology of spatial luminance contrast sensitivity. Psychol. Neurosci. 4, 29–48 10.3922/j.psns.2011.1.005

[B8] Del Bene F. Wyart C. (2012). Optogenetics: a new enlightenment age for zebrafish neurobiology. Dev. Neurobiol. 72, 404–414. 10.1002/dneu.20914 21567983

[B9] Derefeldt G. Lennerstrand G. Lundh B. (1979). Age variations in normal human contrast sensitivity. Acta Ophthalmol. (Copenh) 57, 679–690. 10.1111/j.1755-3768.1979.tb00517.x 525292

[B10] De Valois R. L. De Valois K. K. (1980). Spatial vision. Annu. Rev. Psychol. 31, 309–341. 10.1146/annurev.ps.31.020180.001521 7362215

[B11] Duffy K. R. Hubel D. H. (2007). Receptive field properties of neurons in the primary visual cortex under photopic and scotopic lighting conditions. Vision Res. 47, 2569–2574. 10.1016/j.visres.2007.06.009 17688906 PMC2951600

[B12] Enroth-Cugell C. Robson J. G. (1966). The contrast sensitivity of retinal ganglion cells of the cat. J. Physiol. 187, 517–552. 10.1113/jphysiol.1966.sp008107 16783910 PMC1395960

[B13] Ewert J. P. Buxbaum-Conradi H. Dreisvogt F. Glagow M. Merkel-Harff C. Röttgen A.. (2001). Neural modulation of visuomotor functions underlying prey-catching behaviour in anurans; perception, attention, motor performancs, learning. Comp. Biochem. Physiol. A Mol. Integr. Physiol. 128, 417–461. 10.1016/s1095-6433(00)00333-0 11246037

[B14] Fadool J. M. Dowling J. E. (2008). Zebrafish: a model system for the study of eye genetics. Progr. Retin. Eye Res. 27, 89–110. 10.1016/j.preteyeres.2007.08.002 17962065 PMC2271117

[B15] Fite K. V. Rosenfieldwessels S. (1975). Comparative study of deep avian foveas. Brain Behav. Evol. 12, 97–115. 10.1159/000124142 811324

[B16] Franco L. M. Zulliger R. Wolf-Schnurrbusch U. E. K. Katagiri Y. Kaplan H. J. Wolf S.. (2009). Decreased visual function after patchy loss of retinal pigment epithelium induced by low-dose sodium iodate. Invest. Ophthalmol. Vis. Sci. 50, 4004–4010. 10.1167/iovs.08-2898 19339739

[B17] Gaillard F. Bonfield S. Gilmour G. S. Kuny S. Mema S. C. Martin B. T.. (2008). Retinal anatomy and visual performance in a diurnal cone-rich laboratory rodent, the Nile grass rat (Arvicanthis niloticus). J. Comp. Neurol. 510, 525–538. 10.1002/cne.21798 18680202

[B18] Haug M. F. Biehlmaier O. Mueller K. P. Neuhauss S.C.F. (2010). Visual acuity in larval zebrafish: behavior and histology. Front. Zool. 7:8. 10.1186/1742-9994-7-8 20193078 PMC2848032

[B19] Hess R. F. Nordby K. (1986). Spatial and temporal properties of human rod vision in the achromat. J. Physiol. 371, 387–406. 10.1113/jphysiol.1986.sp015982 3486272 PMC1192731

[B20] Hughes A. (1977). “The topography of vision in mammals of contrasting life style: comparative optics and retinal organisation,” in The Visual System in Vertebrates, ed Crescitelli F. (Berlin: Springer), 613–756.

[B21] Jacobs G. H. Birch D. G. Blakeslee B. (1982). Visual-acuity and spatial contrast sensitivity in tree squirrels. Behav. Processes 7, 367–375. 10.1016/0376-6357(82)90008-0 24923501

[B22] Kelly D. H. (1972). Adaption effects on spatio-temporal sine-wave thresholds. Vision Res. 12, 89–101. 10.1016/0042-6989(72)90139-3 5034636

[B44] Lundh B. L. Lennerstrand G. Derefeldt G. (1983). Central and peripheral normal contrast sensitivity for static and dynamic sinusoidal gratings. Acta Ophthalmol. 61, 171–182. 10.1111/j.1755-3768.1983.tb01410.x 6880630

[B23] Maaswinkel H. Li L. (2003). Spatio-temporal frequency characteristics of the optomotor response in zebrafish. Vision Res. 43, 21–30. 10.1016/s0042-6989(02)00395-4 12505601

[B24] Northmore D. (2010). “Optic Tectum,” in Encyclopedia of Fish Physiology: From Genome to Environment, ed Farrell A. (Londen, UK: Elsevier Academic Press), 131–142.

[B25] Owsley C. (2003). Contrast sensitivity. Ophthalmol. Clin. North Am. 16, 171–177. 10.1016/S0896-1549(03)00003-8 12809156

[B26] Ransomhogg A. Spillmann L. (1980). Perceptive field size in fovea and periphery of the light-adapted and dark-adapted retina. Vision Res. 20, 221–228. 10.1016/0042-6989(80)90106-6 7385595

[B27] Ren J. Q. Mccarthy W. R. Zhang H. Adolph A. R. Li L. (2002). Behavioral visual responses of wild-type and hypopigmented zebrafish. Vision Res. 42, 293–299. 10.1016/s0042-6989(01)00284-x 11809482

[B28] Rinner O. Rick J. M. Neuhauss S. C. (2005). Contrast sensitivity, spatial and temporal tuning of the larval zebrafish optokinetic response. Invest. Ophthalmol. Vis. Sci. 46, 137–142. 10.1167/iovs.04-0682 15623766

[B29] Schade O. H. (1956). Optic and photoelectric analog of the eye. J. opt. Soc. Am. 46, 721–739. 10.1364/josa.46.000721 13358013

[B31] Spillmann L. (2006). From perceptive fields to Gestalt. Prog. Brain Res. 155, 67–92. 10.1016/s0079-6123(06)55005-8 17027381

[B32] Tappeiner C. Balmer J. Iglicki M. Schuerch K. Jazwinska A. Enzmann V.. (2013). Characteristics of rod regeneration in a novel zebrafish retinal degeneration model using N-methyl-N-nitrosourea (MNU). Plos One 8:e71064. 10.1371/journal.pone.0071064 23951079 PMC3741320

[B33] Tappeiner C. Gerber S. Enzmann V. Balmer J. Jazwinska A. Tschopp M. (2012). Visual acuity and contrast sensitivity of adult zebrafish. Front. Zool. 9:10. 10.1186/1742-9994-9-10 22643065 PMC3453526

[B34] Umino Y. Solessio E. Barlow R. B. (2008). Speed, spatial and temporal tuning of rod and cone vision in mouse. J. Neurosci. 28, 189–198. 10.1523/jneurosci.3551-07.2008 18171936 PMC2847259

[B35] van Hateren J. H. (1992). Real and optimal neural images in early vision. Nature 360, 68–70. 10.1038/360068a0 1436076

[B36] van Hateren J. H. (1993). Spatiotemporal contrast sensitivity of early vision. Vision Res. 33, 257–267. 10.1016/0042-6989(93)90163-q 8447098

[B37] Wandell B. A. (1995). Foundations of Vision. Sunderland, Massachusetts: Sinauer Press.

[B38] Wang J. S. Kefalov V. J. (2011). The cone-specific visual cycle. Prog. Retin. Eye Res. 30, 115–128. 10.1016/j.preteyeres.2010.11.001 21111842 PMC3073571

[B39] Warrant E. J. (1999). Seeing better at night: life style, eye design and the optimum strategy of spatial and temporal summation. Vision Res. 39, 1611–1630. 10.1016/s0042-6989(98)00262-4 10343855

[B40] Watson A. (1986). Temporal sensitivity in Handbook of Perception and Human Performance, Vol 1, Sensory Processes and Perception, edS Boff K. R. Kaufman L. Thomas J. P. (New York: Wiley), chapter 6, 1–43.

[B41] West S. K. Rubin G. S. Broman A. T. Munoz B. Bandeen-Roche K. Turano K. (2002). How does visual impairment affect performance on tasks of everyday life? The SEE project. Salisbury eye evaluation. Arch. Ophthalmol. 120, 774–780. 10.1001/archopht.120.6.774 12049583

[B42] Westerfield M. (1995). The Zebrafish Book: A Guide for the Laboratory Use of Zebrafish (Brachydanio Rerio). Eugene, Oregon: University of Oregon Press.

[B43] Wikler K. C. Rakic P. (1990). Distribution of photoreceptor subtypes in the retina of diurnal and nocturnal primates. J. Neurosci. 10, 3390–3401. 2145402 10.1523/JNEUROSCI.10-10-03390.1990 PMC6570186

